# Effect of buccal fat pad, platelet-rich fibrin (PRF) and buccal advancement flap in oroantral fistula closure

**DOI:** 10.6026/9732063002001765

**Published:** 2024-12-31

**Authors:** Asha S Badadesai, Nureldeen AN Elhammali, Abhigyan Manas, Tanu Priya Sonkar, Antara Majumdar, Smita Sutar, Madhvika Patidar, Ashwini Narayankar

**Affiliations:** 1 Department of Oral and Maxillofacial Surgery, PMNM Dental College and Hospital, Bagalkot - 587103, Karnataka, India; 2 Department of Oral and Maxillofacial Surgery, Sirte University, Libya, North Africa; 3 Department of Oral and Maxillofacial Surgery, Babu Banarsi Das College of Dental Sciences, BBD University, Lucknow, India; 4 Department Dentistry (Prosthodontics and Crown & Bridge), Autonomous State Medical College, Firozabad, India; 5 Intern, Kalinga Institute of Dental Sciences, KIIT University, Bhubaneswar, Odisha, India; 6 Department of Oral and Maxillofacial Surgery, Rajesh Ramdasji Kambe Dental College, Akola, Maharashtra, India; 7 Department of Oral Pathology and Microbiology, Government College of Dentistry, Indore, Madhya Pradesh, India; 8 Department of Prosthodontics, SB Patil Dental College, Bidar, Karnataka, India

**Keywords:** Oroantral fistula (OAF), buccal pad of fat (BPF), flap, fistula, management, platelet-rich fibrin (PRF), oroantral communication (OAC)

## Abstract

An oroantral fistula (OAF) is a pathological abnormal communication between the oral cavity and the maxillary sinus which may arise as a result of failure of primary healing of an OAF which can result into the maxillary sinusitis. Therefore, a number of methods are attempted to close this connection using various types of flap or graft. The objective of the research was to evaluate the closure of the oroantral fistula using the buccal advancement flap (BAF), platelet-rich fibrin (PRF) and buccal pad of fat. The study comprised 30 healthy patients with oroantral fistulas. Based on the method utilised to close the OAF, the samples were split into three groups: Group I used a buccal pad of fat, Group II used platelet-rich fibrin and Group III used a buccal advancement flap. All groups showed satisfactory healing, however the BPF and PRF groups experienced much less post-operative pain, clinical healing scores and swelling than the BAF group. BPD and PRF were found to be effective in closing OAF.

## Background:

An aberrant pathologic connection between the maxillary sinus and the oral cavity is known as an oroantral fistula (OAF). Implant surgery, dental extraction, trauma, sinusitis, infection, osteomyelitis and iatrogenic consequences are typically linked to this adverse event [1, 2]. Tooth extractions in the maxillary posterior region are the most frequent causes of oroantral communication (OAC) [3, 4]. It has been commonly observed that persistent connection between the maxillary sinus and the oral cavity can cause maxillary sinusitis by serving as a channel for bacterial and fungal invasion. An oroantral fistula (OAF) may form when an OAC fails to close and the opening becomes epithelialized [5]. Fluid regurgitation and altered nasal resonance can result from an oroantral fistula. It should be noted that OACs might close by itself if the defect is < 3 mm in size. If the size is greater, a flap may be required for surgical closure [6]. OAF closure is difficult and technique-sensitive. There are several methods that can be applied to the closure. The location and size of the defect are the primary determinants of the type of surgery required to close OAF [7]. Numerous treatment approaches, including the use of grafts (allogenous materials, xenografts), advanced flaps, platelet-rich fibrin, buccal pads of fat and synthetic materials, have been developed for the management of OAC and OAF and have demonstrated high rates of effective defect closure [5, 6, 7, 8, 9-10]. Both a clot and a membrane can be formed only from prich fibrin and guided tissue regeneration (GTR) membrane. These methods have the significant benefit of not requiring donor site surgery, which results in significant time and financial savings as well as, more significantly, less patient discomfort [2]. All surgical approaches, however, carry the risk of postoperative discomfort and oedema [5]. The most widely utilised procedure to date is a surgical strategy that combines the palatal rotation-advancement flap technique and a buccal advancement flap (BAF) [6, 8]. Inside the masticatory gaps, the buccal fat pad (BFP) is a lobulated mass of fatty tissue encased in a thin capsule [11]. Regardless of a person's body weight and distribution of body fat, their BFP size stays constant. It is accessible nearby to the surgical site, so it reduces surgical time. It has a great blood supply and is simple to harvest and mobilise. It gets over the drawback of a decrease in vestibular depth that happens when a buccal advancement flap is used for reconstruction [7, 12]. A second-generation platelet concentrate called Platelet Rich Fibrin (PRF) has a high concentration of platelets and growth factors contained in a fibrin matrix. These growth factors and platelets improve the tissues' capacity to repair and reduce inflammatory responses [13]. PRF is a biologically appropriate graft for OAC because of its robust fibrin matrix and advantageous mechanical characteristics, such as flexibility and elasticity. It doesn't trigger allergic reactions because it's an autologous product [6]. Wound healing is positively impacted by PRF [5]. Fibrin, vitronectin and fibronectin are secreted by platelets and serve as adhesion molecules for cell migration, growth factors that promote the development of stem cells, fibroblasts, osteoblasts and the extracellular matrix [12]. Therefore, it is of interest to evaluate the oro antra fistula closure using buccal pad of fat, platelet-rich fibrin and buccal advancement flap.

## Materials and Methods:

The present single-centred, prospective, randomized follow-up study was done in the Oral and Maxillofacial Surgery Department after obtaining ethical clearance from concerned authority and informed consent from all the participants. Healthy 30 individual who visited to Oral and Maxillofacial OPD having oroanatral fistula were included for the study. The samples were arbitrarily alienated into 3 groups based on the technique used for closure of OAF; Group I: using buccal pad of fat, Group II: using platelet rich fibrin and Group III: using buccal advancement flap. Every patient had a thorough medical history and a clinical assessment. The general health of the patient was evaluated. A traditional blunt probe was used in the initial clinical examination to determine the diagnosis of OAC. To determine whether the size of the perforation was greater than 3 mm, the Valsalva test was conducted and measurements were taken of the OAC's diameter, site and duration. The surgical closer of OAF was done with either of 3 methods.

## PRF technique:

10 millilitres of the patient's venous blood were used to make PRF. The wound was thoroughly cleaned and sanitised. The mucoperiosteal flap was raised around 5 mm around the tooth socket's edge. A PRF clot was thickly packed inside the alveolus or bone socket and it was encased in another PRF membrane. The surrounding muco-periosteal flap was sutured to the PRF membrane that covered the clot using 4-0 vicryl.

## Buccal advancement flap (BAF):

The wound was meticulously cleaned and sanitised under local anaesthetic. This method involved creating a broad-based trapezoid mucoperiosteal flap that extended from the buccal sulcus to the tooth socket's edge. The flap was sutured over the defect after being reflected and advanced. A basal periosteal releasing incision was made parallel to the coronal margin in order to close the wound without creating strain. The periosteum, connective tissue and epithelium made up this trapezoidal flap.

## Buccal pad of fat method (BPF):

A 2-cm horizontal periosteal incision that extended rearward above the maxillary second molar tooth and was lateral to the maxillary buttress exposed the BFP. After being gently pushed out and mobilised, the buccal extension and BFP were carefully advanced into the bone defect and sutured. Every patient received instruction about maintaining good oral hygiene after surgery, eating a soft diet and restriction of physically activities. Patients were instructed not to blow their nostrils for 21 days following surgery. The displacement of the mucogingival line (MGL), the postoperative pains score (VAS) and the defect closure healing process was assessed during a 21-day prospective follow-up. Clinical Healing Scores (CHS) were used to quantify the inflammatory response and the wound healing process clinically. The obtained data was statistically evaluated using SPSS statistical software version 22.0 using ANOVA and post-hoc test and p<0.05.

## Results:

## Post-operative pain score:

When compared to the BAF group, the BPF and PRF groups showed better recovery and less post-operative pain and swelling. BPD and PRF were found to be effective in closing OAF (Table 1).

## Displacement of MGL:

Table 2 displays the clinical displacement of the mucogingival line at baseline (preoperatively), on day 7 and on day 21 in the PRF, BPF and BAF groups. Significantly lower values after one week and 21 days (p < 0.001) were sea in the BAF group. There were no post-hoc significances found for the PRF and BPF groups.

## Clinical healing scores:

Through the presence or absence of oedema, redness, healthy granulation tissue and epithelisation on the surgical site, CHS is used to evaluate the inflammatory response and the healing process. The findings showed that Groups 1 and 2 had substantially lower CHS than Group 3. According to Table 3, the P value was statistically significant as well (Table 3).

## Discussion:

An abnormal epithelialized passage between the maxillary sinus and oral cavity is known as an oroantral fistula (OAF). Excruciating pain, nasal fluid leakage, airflow from the mouth into the nose, epistaxis, a change in voice due to resonance and purulent discharge are all symptoms of chronic OAF [7]. Since OAC vary in size, a variety of treatments are available. Soft tissue covering is thought to be the most crucial factor in OAC healing [12]. The dislocation of the mucogingival borderline also showed a considerable disparity between the three approaches in terms of the assessment of the secondary outcome measurements. After the buccal advancement flap treatment, the mucogingival boundary was expected to shift; nevertheless, the platelet-rich fibrin and BPF groups also had a minor displacement of the mucogingival borderline. This could be explained by the remodelling process brought on by the extraction sockets' bundle bone resorption. It is normal to experience mild to severe pain following a minor surgical operation. Over the course of the follow-up period, no patient reported excruciating pain. In comparison to the advanced flap approach, we reported lower pain scores in PRF and BPF. According to the results of this study, there were substantial differences between the groups in the presence of pain and the amount of medications that were required. This is in association with the research conducted by Bilginaylar *et al.* [14]. We found that both PRF membrane and BFP are effective in the management of OAF in present study. Mahmoud evaluated the Buccal Pad of Fat versus Leukocyte Platelet Rich Fibrin (L-PRF) in the management of oroantral communication. He found considerable variation between the two groups regarding pain and swelling [12]. In comparison to the buccal fat pad, Hariram *et al.* found that the sandwich graft approach produced a better closure of oroantral communication by offering a more biologically appropriate substrate in terms of regeneration of damaged bone structure at the maxillary sinus floor [9]. Our study's findings concurred with those of Dolanmaz *et al.* who determined that pedicled BFP flaps are a suitable and trustworthy substitute for treating acute or chronic oroantral communication [8]. Nezafati *et al.* evaluated the clinical outcomes of the regular buccal flap and the pedicled buccal fat pad flap (PBFPF) for the closure of oro-antral fistula. They came to the conclusion that the experimental group's pain score was statistically higher and that both approaches were equally effective in closing OAF [15]. The effectiveness of buccal advancement pads and buccal fat pads for closing oroantral fistulas was assessed by Shukla *et al.* Despite its higher morbidity, they came to the conclusion that buccal fat pads were a preferable option for OAF closure [7]. Hunger *et al.* assessed the effectiveness of buccal advancement flaps (BAF) or platelet-rich fibrin (PRF) clots in closing oroantral connections. They came to the conclusion that using PRF clots to fill defects is linked to decreased discomfort and mucogingival boundary displacement [5]. In order to assess the effectiveness of Platelet-Rich Fibrin (PRF) for the closure of acute OAC, Awal *et al.* compared it with buccal advancement flap. The group treated with PRF clot showed statistically significant improvements in healing with fewer problems [6]. PRF clot and membrane preparation is incredibly quick and uncomplicated and it can be completed quickly. The current study's limitations include a single-center design, a smaller sample size and an OAC size of less than 8 mm. More research with a bigger sample size is required. A limited sample size, a single-center study and an OAC size of less than 8 mm are the study's limitations. Larger sample sizes require further research.

## Conclusion:

Data shows that all groups have satisfactory healing. However, the BPF and PRF groups experienced much less post-operative pain, clinical healing scores and swelling than the BAF group. It is very easy and simple to prepare PRF clot and membrane. Further, it can be put together quickly.

## Figures and Tables

**Table 1:** Post-operative pain

**Duration**		**Technique**		**p**
	**Buccal pad of fat**	**Platelet rich fibrin**	**Buccal advancement flap**	
Day 1	4.03	3.06	7.51	
Day 7	2.43	2.12	6.65	0.001
Day 14	0.32	0.21	5.32	

**Table 2:** Clinical dislocation of the mucogingival line (MGL)

**Duration**		**Technique**		**p**
	**Buccal pad of fat**	**Platelet rich fibrin**	**Buccal advancement flap**	
Baseline	9.2±1.8	8.2±1.7	9.0±1.6	0.432
After 1 week	9.2±1.7	2.3±1.5	8.8±1.5	0.001
After 21 days	8.7±1.6	1.6±1.9	8.4±1.6	0.001

**Table 3:** Clinical healing scores

**Technique**	**CHS score Mean±SD**	**p**
Buccal pad of fat	6.65±1.34	
Platelet rich fibrin	5.23±0.14	0.003
Buccal advancement flap	5.42±0.56	

## References

[1] Kwon M.S (2020). J Korean Assoc Oral Maxillofac Surg..

[2] Parvini P (2018). International Journal of Implant Dentistry..

[3] Hassan O (2012). Egypt J Ear Nose Throat Allied Sci..

[4] Awang M.N (1988). Int J Oral Maxillofac Surg..

[5] Hunger S (2023). Clinical Oral Investigations..

[6] Awal M.A (2020). Biomed J Sci & Tech Res..

[7] Shukla B (2021). Natl J Maxillofac Surg..

[8] Dolanmaz D (2004). Quintessence international..

[9] Hariram (2010). Natl J Maxillofac Surg..

[10] Batra H (2010). J Maxillofac Oral Surg..

[11] Visscher S.H (2010). J Oral Maxillofac Surg..

[12] Mahmoud N.R (2020). Egyptian Dental Journal..

[13] Dohan D.M (2006). Oral Surgery Oral Medicine Oral Pathology Oral Radiology and Endodontics..

[14] Bilginaylar K (2019). J Craniofac Surg..

[15] Nezafati S (2012). Int J Oral Maxillofac Surg..

